# Effect of B_2_O_3_ on the Structure, Properties and Antibacterial Abilities of Sol-Gel-Derived TiO_2_/TeO_2_/B_2_O_3_ Powders

**DOI:** 10.3390/ma16196400

**Published:** 2023-09-25

**Authors:** Albena Bachvarova-Nedelcheva, Reni Iordanova, Angelina Stoyanova, Nelly Georgieva, Veronica Nemska, Tsvetelina Foteva

**Affiliations:** 1 Institute of General and Inorganic Chemistry, Bulgarian Academy of Sciences, Acad. G. Bonchev Str., bld. 11, 1113 Sofia, Bulgaria; reni@svr.igic.bas.bg; 2 Department Chemistry and Biochemistry, Faculty of Pharmacy, Medical University—Pleven, Kl. Ohridski Str., 1, 5800 Pleven, Bulgaria; angelina.stoyanova@mu-pleven.bg; 3 Department Biotechnology, Faculty of Chemical and Systems Engineering, University of Chemical Technology and Metallurgy, Kl. Ohridski Blvd, 8, 1756 Sofia, Bulgaria; nelly.georgieva@yahoo.com (N.G.); vnemska@uctm.edu (V.N.); tsvetelina_angelova@uctm.edu (T.F.)

**Keywords:** sol-gel, IR, UV-Vis spectra, photocatalysis, antibacterial properties

## Abstract

This paper studies the influence of B_2_O_3_ on the structure, properties and antibacterial abilities of sol-gel-derived TiO_2_/TeO_2_/B_2_O_3_ powders. Titanium(IV) butoxide, telluric(VI) acid and boric acid were used as precursors. Differences were observed in the degree of decomposition of Ti butoxide in the presence of H_3_BO_3_ and H_6_TeO_6_ acids. The phase transformations of the obtained gels in the temperature range of 200–700 °C were investigated by XRD. Composite materials containing an amorphous phase and different crystalline phases (metallic Te, α-TeO_2_, anatase, rutile and TiTe_3_O_8_) were prepared. Heating at 400 °C indicated a crystalline-to-amorphous-phase ratio of approximately 3:1. The scanning electron microscopy (SEM) analysis showed the preparation of plate-like TiO_2_ nanoparticles. The IR results showed that the short-range order of the amorphous phases that are part of the composite materials consists of TiO_6_, BO_3_, BO_4_ and TeO_4_ structural units. Free B_2_O_3_ was not detected in the investigated compositions which could be related to the better connectivity between the building units as compared to binary TiO_2_/B_2_O_3_ compositions. The UV-Vis spectra of the investigated gels exhibited a red shift of the cut-off due to the presence of boron and tellurium units. The binary sample achieved the maximum photodegradation efficiency (94%) toward Malachite green dye under UV irradiation, whereas the ternary sample photoactivity was very low. The compositions exhibited promising antibacterial activity against *E. coli* NBIMCC K12 407.

## 1. Introduction

Boron oxide (B_2_O_3_) is a well-known classical glass former, and the sol-gel method is not very often used for the synthesis of amorphous borate materials [1]. Within the last two decades, there has been increased interest in B_2_O_3_-containing compositions due to their unique properties and great promise for engineering applications [2,3,4]. For example, borate glasses are less chemically durable due to their bonding and structure and are therefore more bioactive. It is well known that numerous investigations focused mainly on the sol-gel processes for silica-based compositions have been published. Up to now, only a limited number of studies on borate glasses created through the sol-gel process [4] have appeared. Recently, borate compositions obtained by using the sol-gel method have been intensively studied due to their promising bioactive properties [4]. 

The first gel-derived borate materials are reported in the systems: B_2_O_3_-Li_2_O [5] and B_2_O_3_-BaO [6]. Borosilicate glasses obtained by using the sol-gel method with a more homogeneous network compared to the traditional melting route were reported [7,8,9,10,11]. Some authors found that the short-range order of the alkaline borate gels and multicomponent borosilicate gels closely resembles that of the bulk melt-quenching glasses [12,13]. The positive role of boron ions was proven in the bioactivity of the sol-gel prepared SiO_2_/P_2_O_5_/CaO/B_2_O_3_ samples [14]. The multi-component hybrid materials have been fabricated, and it is shown that the nature of the organic group plays a major role in the design of new sol-gel materials [15]. The as-prepared by sol-gel technology B_2_O_3_/Na_2_O/TiO_2_ gel region corresponds fairly well to the twin-roller glass region. Up to now, the connectivity between borate and titanate units is questionable, and as it is known, boro-titanate glasses are not obtained from melts. The incorporation of boron into the TiO_2_ bulk extends its visible light absorption [16,17]. The photoactivity of TiO_2_-B_2_O_3_ and B_2_O_3_-SiO_2_/TiO_2_ catalysts was improved by the boron content [18,19]. 

As an intermediate oxide, TiO_2_ does not form glass by conventional quenching but successfully is used to produce amorphous and crystalline materials via the sol-gel process [20,21]. It was found that the addition of TiO_2_ to TeO_2_ allows for enhancing the glass-forming ability and obtaining homogeneous glasses [22,23]. Moreover, it has been found that the positive role of TiO_2_ is to preserve the glass network of pure TeO_2_ which contributes to the optical properties of these materials [24]. On the other hand, sol-gel-derived titania products are extensively used as catalysts, supports, protective coatings, self-cleaning surfaces and as an active ingredient of sunscreen cosmetic products [20].

It is well known that tellurite glasses possess a low melting point and the absence of hygroscopic properties [25,26]. Thus, it is useful to search for new reproducible routes for synthesis in order to extend their applications. The sol-gel technique is a low-temperature alternative that has been developed and is suitable for the investigation of new tellurite compositions.

Sol-gel obtaining of different binary, ternary and multicomponent composite powders containing TiO_2_ is one of the main tasks developed by our team. Up to now, we have determined the gel formation regions in several binary and ternary systems [27,28,29,30,31]. Investigations on the structure, thermal, optical, photocatalytic and antibacterial properties of selected compositions have been performed [32,33,34,35]. Moreover, the obtained results on the photocatalytic and antibacterial properties of some nanosized powders were promising for their environmental applications [32,33,34,35]. The present study extends our investigations on the sol-gel obtaining of TiO_2_ nanocomposite powders by applying Te(VI) acid and Ti(IV) n-butoxide as a combination of precursors. The novelty of this work was studying the influence of B_2_O_3_ on the structure, properties and antibacterial abilities of sol-gel-derived TiO_2_/TeO_2_/B_2_O_3_ powders as well as verifying their biocidal and environmental applications. The lack of information in the literature concerning this problem additionally emphasizes its recency.

## 2. Materials and Methods

### 2.1. Gelling and Drying

Based on our previous findings on the gel formation in the ternary TiO_2_-TeO_2_-B_2_O_3_ system, compositions containing higher TiO_2_ content were selected—50TiO_2_.25TeO_2_.25B_2_O_3_, 80TiO_2_.10TeO_2_.10B_2_O_3_ and 80TiO_2_.20B_2_O_3_, denoted as samples A, B and C, respectively. They were subjected to detailed investigations. The gels were prepared using a combination of Te(VI) acid (Aldrich, St. Louis, MO, USA) along with Ti butoxide (Fluka AG, Everett, WA, USA) and H_3_BO_3_ (Merck, Rahway, NJ, USA) as precursors dissolved in ethylene glycol (C_2_H_6_O_2_) (99% Aldrich). Telluric acid (H_6_TeO_6_) was selected to overcome the problem of the high hydrolysis rate of tellurium(VI) alkoxide that has been analyzed in several papers [36,37]. The precursor solutions were subjected to 5–10 min intensive stirring at room temperature to achieve complete dissolution. No direct addition of water was made to the precursor solutions. Sol-gel hydrolysis reaction was acquired from absorbed atmospheric moisture. The measured pH was 4–5 depending on composition. The gelation time for the investigated compositions occurred immediately. For completing the hydrolysis, the aging of gels was performed in air for several days. The obtained gels were subjected to stepwise heating from 200 to 700 °C for one hour of exposure time in the air. Aiming to verify the phase and structural transformations of the gels, the heating in the range of 200–700 °C until obtaining powders was performed. The selection of the temperatures was made based on our previous investigations [27,28,29,30,31]. 

### 2.2. Sample Characterization

Powder XRD patterns were registered at room temperature with a Bruker D8 Advance (Berlin, Germany) X-ray powder diffractometer with a Cu Ka radiation (k = 1.54056 Å) and a LynxEye solid position sensitive detector with X-ray tube operated at 40 kV and 40 mA. X-ray diffraction patterns were recorded in the range of 5.3–80° 2 h with a step of 0.02° 2 h. The differential thermal analysis (LABSYSTM EVO apparatus, Setaram, Lyon, France) with Pt-Pt/Rh thermocouple at a heating rate of 10 K/min in air flow and Al_2_O_3_ as a reference material was used to study the decomposition process of the gels. The accuracy of the temperature was ±5 °C and the heating of the samples was limited up to 700 °C. Gases evolved (EGA) during the thermal treatments were analyzed by mass spectrometry (MS) with a Pfeiffer OmniStarTM mass spectrometer (Pfeiffer Vacuum Technology AG, Wetzlar, Germany). Mass spectra recorded for investigated samples show the *m/z* = 14, 18 and 44 signals, attributed to CH_2_, H_2_O and CO_2_, respectively. The infrared spectra were made in the range of 1600–400 cm^−1^ using the KBr pellet technique on a Nicolet-320 FTIR spectrometer (Madison, WI, USA) with 64 scans and a resolution of ±1 cm^−1^. The UV–VIS diffused reflectance Spectrophotometer Evolution 300 (Thermo Electron Corporation, Madison, WI, USA) with a magnesium oxide reflectance standard as the baseline was used for recording the optical absorption spectra of the powdered samples in the wavelength range 200–800 nm. Planck’s equation was applied for the absorption edge and optical bandgap (Eg) determination [31,32,33]. The as-obtained samples were imaged by using a scanning electron microscope (SEM) JSM-5510 (JEOL, Akishima shi, Japan), operated at 10 kV of acceleration voltage. The investigated samples were coated with gold by using JFC-1200 fine coater (JEOL) before observation. The specific surface areas (BETs) were determined by low-temperature (77.4 K) nitrogen adsorption in NOVA (Osaka, Japan) 1200e surface area & pore analyzer at relative pressures p/p0 = 0.1–0.3 using the BET equation.

#### 2.2.1. Photocatalysis

The photocatalytic activity of the investigated pure and composite titania powders was evaluated by UV-light induced photobleaching of Malachite green (MG) dye solution, used as a representative dye pollutant. The MG working solutions of concentration 5 ppm were prepared by serial dilution of 0.1 mol/L stock solution of the dye with bi-distilled water. The procedure was as follows: 100 mg sample was immersed in 150 mL dye solution. The resulting suspension was stirred in the dark for 30 min to obtain MG adsorption equilibrium before irradiation. A black-light blue UV lamp (Sylvania BLB 50 Hz 8W T5) was used as a source of UV light with a main emission wavelength of 365 nm. The lamp was positioned at a distance of 10 cm from the solution surface. All photocatalytic tests were performed at room temperature and constant stirring (450 rpm). At certain time intervals of illumination, 3 mL of the suspensions was withdrawn and centrifuged at 5000 rpm for 10 min to separate the supernatant (containing MG dye with a certain concentration) from the solid particles. Then, the concentration changes of MG dye were monitored by measuring the absorbance of clear aliquots using a Jenway 6505 UV-Vis spectrophotometer (designed and manufactured in Chelmsford, Essex, CM1 3UP, Chelmsford, UK) at 618 nm, the maximum absorption wavelength for MG. As stated in Beer’s law, the absorbance at 618 nm increases linearly with increasing concentration of MG, and the degradation ratio of the dye, C/C_o_, is represented by the ratio A/A_o_, where A_o_ is the absorbance of MG dye solution at time zero, and A is the solution absorbance at a time t. The photocatalytic properties of the composite sample powders were compared with those of a pure TiO_2_ synthesized from Ti(IV) butoxide. 

#### 2.2.2. Antibacterial Properties

##### Test Microorganisms, Media and Culture Conditions

The bacterial strain Escherichia coli NBIMCC K12 407 was supplied by the National Bank for Industrial Microorganisms and Cell Cultures (NBIMCC, Sofia, Bulgaria) and was cultured in Luria-Bertani (LB) broth in a shaker-incubator ES-20/60 (Biosan, Riga, Latvia, 120 rpm) at 37 °C for 24 h.

##### Bactericidal Effect of the Investigated Materials

The potential ability of materials to exhibit growth-inhibiting effects on *E. coli* K12 407 was examined using an agar-well diffusion test. This method consists of the formation of a clear bacterial zone around the materials which are in the form of powder. For this purpose, 100 μL of bacterial strains with a concentration of 1 × 10^7^ CFU/mL, corresponding to 0.5 McFarland, was spread in LB agar plates. After 30 min, the culture was absorbed into the agar plates, and onto the marked wells was placed 100 mg of the investigated materials. The petri dishes were incubated at 37 °C for 24 h. The bactericidal effect of the samples was evaluated by measuring the size of the formed inhibition zones [38]. Three replicates were made from each sample, and the results show the mean values.

In addition, the percent of the bacteria cell reduction by tested materials was calculated. For this aim, 100 μL bacterial suspension with a turbidity of 0.5 McFarland was poured in flasks with 100 mL LB broth and 10 mg of each material. For control, a flask, which contained only bacteria and LB broth, was used. The incubation conditions were shaking at 120 rpm and 37 °C for 24 h. On the next day, 100 μL diluted suspension of each flask was spread on agar plates, and after incubation, the grown colonies were counted, and the percent reduction was calculated according to Bachvarova-Nedelcheva et al. [34]. All tests were performed in triplicates, and the results obtained showed the mean values. 

## 3. Results and Discussion

### 3.1. Phase Formation Studies and Thermal Stability of the Gels

**Figure 1:**
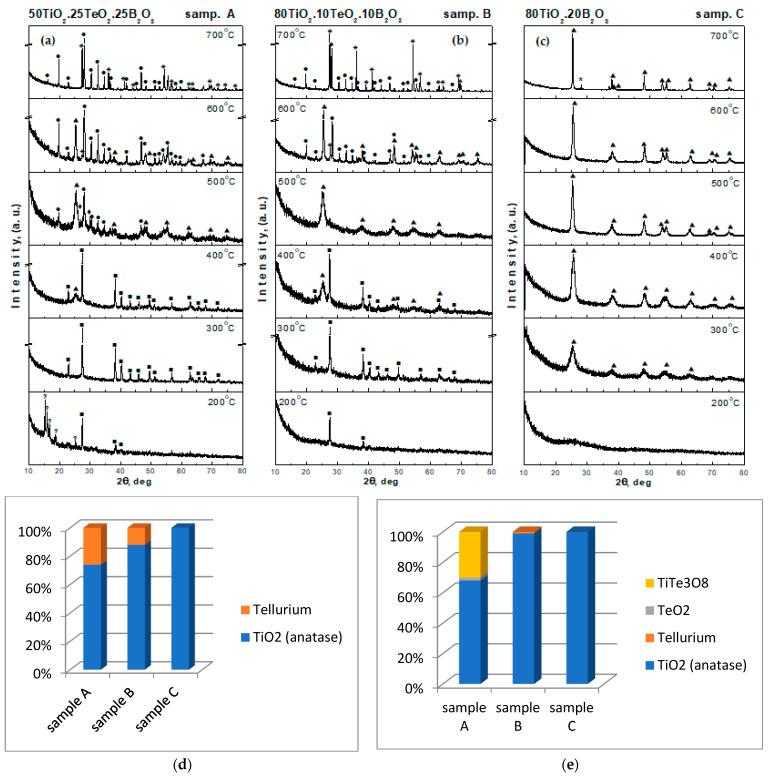
XRD patterns of the investigated samples heat-treated at different temperatures: (■) Te, (♦) α-TeO_2_, (▲) TiO_2_-anatase, (*) TiO_2_-rutile, and (●) TiTe_3_O_8_ (**a**–**c**). Crystalline phase weight content of the studied samples at 400 (**d**) and 500 °C (**e**).

The investigated compositions were situated in the gel formation region in the TiO_2_-TeO_2_-B_2_O_3_ ternary system, which has been investigated previously and published elsewhere [28]. All gels were transparent. The X-ray diffraction patterns of all heat-treated gels are shown in Figure 1a–c. Although samples A and B contained different TiO_2_ amounts, their behavior was very similar up to 400 °C. As can be seen, after heat treatment at lower temperatures (200–300 °C), predominantly an amorphous phase and formation of metallic tellurium only (JCPDS 78-2312) were registered. The amorphous phase amount gradually decreased with increasing the temperature. Tellurium was fully oxidized to TeO_2_ at about 500 °C. First TiO_2_ (anatase) crystals (JCPDS 78-2486) for samples A and B appeared at 400 °C. At this temperature, the crystalline-to-amorphous-phase ratio was approximately 3:1. A small amount was converted to rutile (JCPDS 21-1276) in sample B (containing 80 mol% TiO_2_) at 500 °C, and in the other ternary sample A, rutile was registered at 600 °C. A new crystalline-phase TiTe_3_O_8_ (JCPDS 50-0250) was found at 500 °C for sample A (50 mol% TiO_2_), while in sample B, it appeared at 600 °C. At 500 °C, the amount of the amorphous phase strongly decreased, and it was below 10%. The heat treatment at 400 and 500 °C revealed several crystalline phases which were detected and quantified (Figure 1d,e). Additional heating was performed at 700 °C to complete the phase formation. The coexistence of TiO_2_ (rutile) and TiTe_3_O_8_ was observed in the prepared powders. The behavior of the binary sample C (80TiO_2_.20B_2_O_3_) was different. The amorphous state was preserved up to 300 °C, and further heating of the sample showed the presence of the TiO_2_ (anatase) crystalline phase only. The first TiO_2_ (rutile) crystals appeared at 700 °C. It has to be noted that in all investigated compositions, H_3_BO_3_ was not detected. At 400 °C, the average crystallite size (calculated using Sherrer’s equation) of TiO_2_ (anatase) in the powdered sample C was about 10 nm (Figure 1). The obtained results on the phase formation are similar to those reported by other authors [39,40]. 

**Figure 2:**
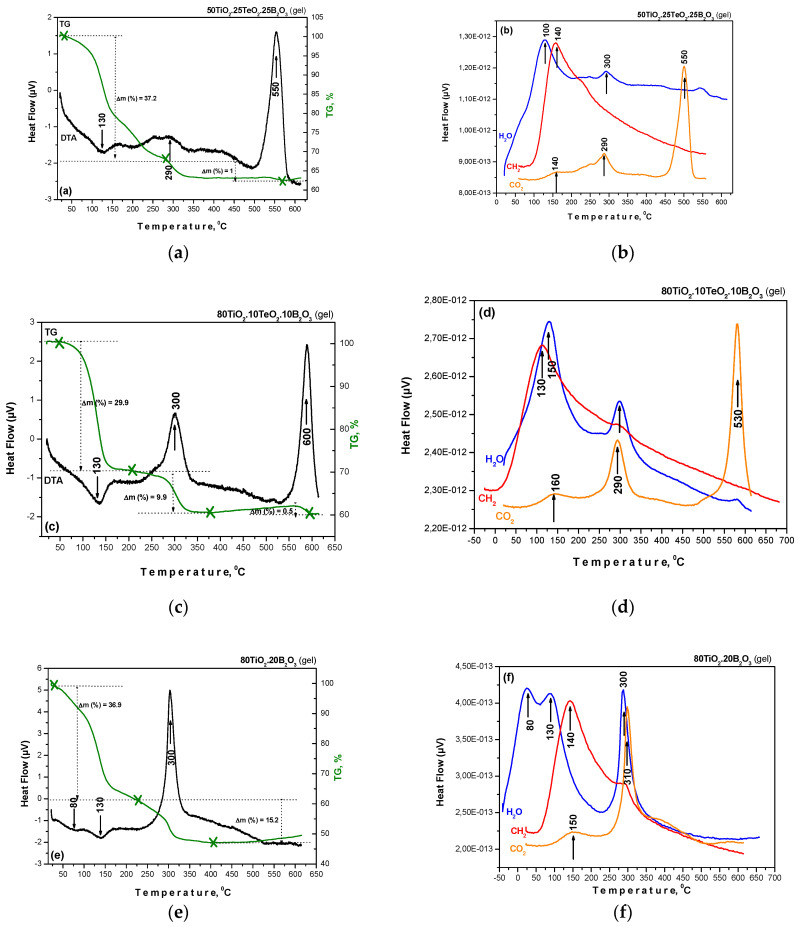
DTA/TG curves (**a**,**c**,**e**) and mass spectra (**b**,**d**,**f**) of the investigated gel compositions.

The thermal decomposition data in the air of the investigated gels are illustrated in Figure 2a–f. Several decomposition steps were observed in the DTA/TG curves of the samples. For the interpretation of these results, DTA data for pure Ti(IV) n-butoxide discussed in the literature [41,42] were taken into consideration. The DTA curves of all gels were characterized by an endothermic peak in the range of 100–200 °C while the binary composition (sample C, Figure 2e) exhibited an additional endothermic effect of about 80 °C. The weight loss was between 30 and 37% depending on composition, and it was related to the elimination of water bonded with the material surface as well as to the evaporation of the organic solvent. In the temperature range of 200–600 °C, several exothermic effects could be distinguished. The first one was detected at about 290–300 °C for all gels, but it was observed that this peak was sharper and more intensive for the binary sample. This could be related to the intense decomposition mainly of Ti(IV) butoxide. In all cases, this peak was accompanied by a weight loss of about 10–15% which according to the TG curve finished at nearly 450 °C. The last exothermic effect, which was stronger for the ternary compositions, was observed in the range of 530–600 °C. It could be attributed to the crystallization of TiO_2_ (anatase) or other crystalline phases. That peak was accompanied by a very low weight loss (~1%), and the calculated total weight loss for the investigated gels was about 45%.

### 3.2. SEM Morphology

**Figure 3:**
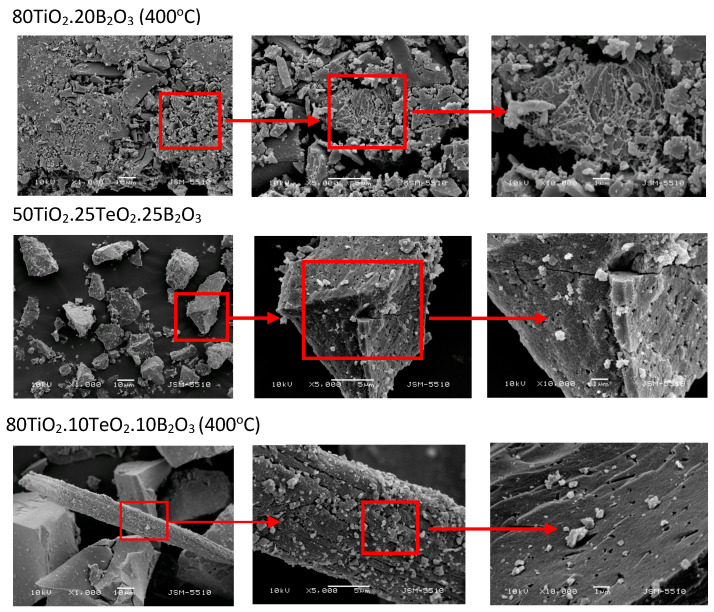
SEM images at different magnifications of the investigated samples heat-treated at 400 °C.

The morphology of the powders was examined by SEM. Figure 3 shows the micrographs at different magnifications of the powders calcined at 400 °C. As seen from the images, the morphology was a result of the crashing of the monolithic gels during the heating. Most of the particles exhibited plate-like or irregular shapes. A strong trend of agglomeration was also observed, and the average size of the aggregates was above 10 μm. Additional confirmation for the agglomeration of the samples was the BET measurements which showed that the specific surface area of the samples was about 35–45 m^2^/g. The microprobe analysis showed no presence of carbon in all samples. Thus, the organic constituents were completely removed at these temperatures. 

Our results confirmed that the particles obtained by using the sol-gel method exhibited strong agglomeration and less porous structure due to the lack of surfactant used in other synthesis methods which usually helps to reduce agglomeration and the size of the nanoparticles [43]. 

### 3.3. Structural Change IR and UV-Vis Spectra

#### 3.3.1. IR Investigations

**Figure 4:**
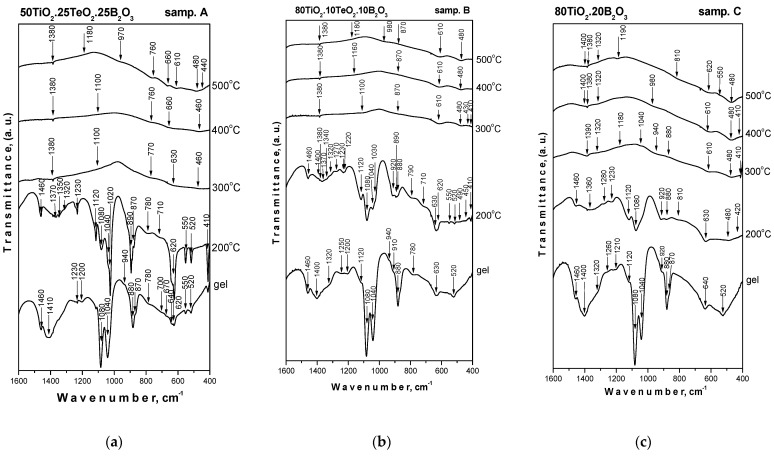
IR spectra of investigated samples heat-treated at different temperatures. (**a**,**b**) samples A and B; (**c**) sample C.

The IR spectra of the investigated composition system heated at different temperatures are shown in Figure 4. The assignments of the vibration bands were made based on spectral data for the precursors, crystalline phases existing in the system and our previous structural investigations on gels in different binary and ternary TiO_2_-containing systems [27,28,29,30,31,32,33,34,35]. As seen in the figure, at low temperatures (up to 200 °C), all gels exhibited similar behavior in the spectral range of 1600–400 cm^−1^. Looking at the spectra, several regions can be distinguished: 1500–1200 cm^−1^, 1200–900 cm^−1^ and 900–400 cm^−1^. By analogy with our previous paper, the IR bands located between 1500 and 1300 cm^−1^ could be assigned to the bending vibrations of CH_3_ and CH_2_ groups [41,44]. It is known that bands in the range of 1130–1040 cm^−1^ are related to the characteristic Ti–O–C stretching vibrations that can be used to determine the degree of hydrolysis [41,44,45]. The presence of unhydrolyzed organic groups (bands at 1120, 1080 and 1040 cm^−1^) and OH groups in the IR spectra of all compositions contributed to the formation of a mixed organic–inorganic amorphous structure. The intensity of absorption bands in the 1120–1040 cm^−1^ region (Ti–O–C stretching vibrations) decreased in the following order: sample A > sample B > sample C. The lowest intensity of these bands was in the spectrum of 80TiO_2_.20B_2_O_3_ (sample C) as compared to the ternary compositions which indicates that borate units are incorporated in the titanate network and lead to a greater degree of hydrolysis at 200 °C. Obviously, the decrease in the TiO_2_ content hinders the hydrolysis–condensation processes as was observed in the spectrum of sample A. 

It has to be noted that in all the gels studied, the TiO_2_ content was higher than that of B_2_O_3_ and TeO_2_ content. Therefore, the vibrational modes observed in the range below 700 cm^−1^ were mainly due to the titanate network. The characteristic vibrations of TiO_6_ structural units in TiO_2_ (anatase) were at 640 and 450 cm^−1^ [20,45,46]. The bands in the range of 700–670 cm^−1^ observed in the spectra of samples A and B could be related to the symmetric TeO_3_ units [47], while deformed TeO_4_ groups exhibited a strong absorption band at 635 cm^−1^ and a shoulder at 670 cm^−1^. On the other hand, in the same absorption range (700–600 cm^−1^), the vibrations of symmetric TeO_6_ units from H_6_TeO_6_ acid were situated [48,49], while bending vibrations of Te–OH were at 1220 and 1130 cm^−1^ [49]. According to the literature data, vibrational modes of different borate units in binary glasses are 1500–1200 cm^−1^ (B–O stretching for trigonal BO_3_ units), 1200–850 cm^−1^ (B–O stretching for tetrahedral BO_4_ units) and 800–600 cm^−1^ (bending vibrations for various borate segments) [50,51,52]. The strong absorption bands at 1480 and 1200 cm^−1^ could be assigned to the presence of B–OH bending vibrations [53,54]. Looking at the spectra, it could be seen that the bands above 1000 cm^−1^, characteristic of the organic groups, were not visible above 300 °C (Figure 4). This could be explained by the transformation of the amorphous phase from organic to inorganic which started at 300 °C. The spectra in the temperature range of 300–500 °C were characterized mainly by bands below 900 cm^−1^, typical for the inorganic units. These bands are with low intensity and broadened, which is a peculiarity of the disordered systems. In both ternary compositions, the weak bands at 770–760 cm^−1^, 660 cm^−1^ and 630–610 cm^−1^ can be assigned with the vibrations of TeO_4_ units [24]. By analogy with our previous IR investigations, bands in the region 700–400 cm^−1^ are characteristic of the formation of a Ti–O–Ti network [28,55,56,57]. The overall temperature range (300–500 °C) with the IR spectrum of all samples exhibited absorption bands centered at 1380 and ~1200 cm^−1^ due to the vibrations of BO_3_ units [58,59,60]. The weak bands about 1100–980 cm^−1^ and 880 cm^−1^ could be associated with the vibrations of B–O bonds in BO_4_ groups. Based on these results, it could be summarized that at higher temperatures, both tellurium and titanium lead to partial BO_3_→BO_4_ transformations in the investigated ternary compositions (Figure 4). The results obtained correlate well with the XRD data already discussed above as well as with our preceding investigations on the sol-gel obtaining of TiO_2_-containing compositions.

#### 3.3.2. UV-Vis Investigations

**Figure 5:**
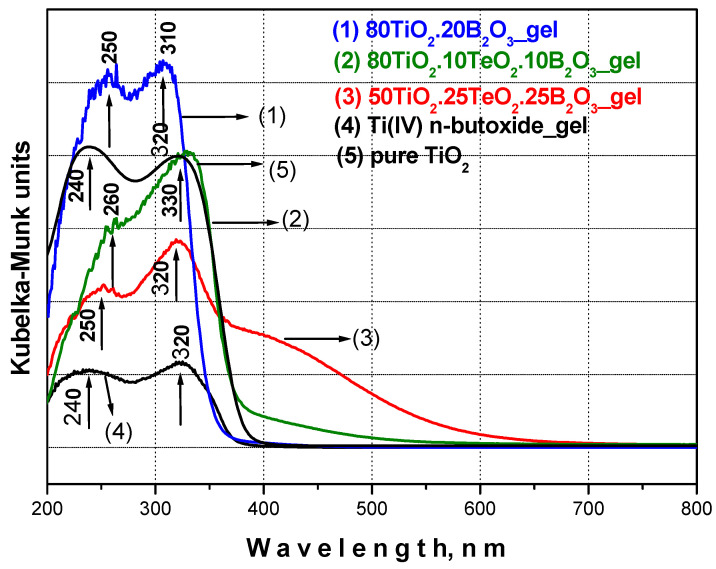
UV-Vis spectra of investigated samples compared with Ti(IV) butoxide gel and pure TiO_2_.

UV-Vis spectroscopy was used as a tool to obtain useful information for the completeness of the hydrolysis–condensation reactions as well as to accumulate additional structural data for the investigated powders. The UV-Vis spectra of the prepared gels are depicted in Figure 5, and they are compared to those of Ti(IV) butoxide gel as well as pure TiO_2_. As seen from the figure, all spectra in the wavelength region from 200 to 800 nm are characterized by two bands 240–260 nm and 310–330 nm, and their UV-Vis spectra are compared to those of TiO_2_ obtained by Ti(IV) butoxide. As has been already discussed in our previous papers, the main building units in the unhydrolyzed Ti butoxide are isolated TiO_4_ groups with absorption bands in the region 240–260 nm [27,31] due to the ligand-to-metal charge transfer. The polymerization processes transformed the TiO_4_ groups into TiO_6_ units possessing absorption at higher wavelengths as the charge transfer in TiO_6_ groups is about 320–340 nm [27,28,61]. 

Bearing in mind the observed UV-Vis data, it could be generalized that all investigated samples exhibited a stronger absorption band at 330–310 nm, which could be related to the higher number of TiO_6_ structural units and consequently more completed hydrolysis–condensation reactions. The other peculiarity is the red shifting toward higher wavelengths. As seen from the figure, sample B (80TiO_2_.10TeO_2_.10B_2_O_3_) exhibited the highest value of the cut-off (431.09 nm). Based on these results, it could be suggested that sample B possessed good antibacterial and/or photocatalytic properties, which will be discussed below. A stronger absorption above 400 nm was observed only in the spectra of sample A (Figure 5). By analogy with our previous paper [27], this phenomenon could be related to carbon which is responsible for visible light absorption. This is indirect proof for the slow processed hydrolysis–condensation processes in this ternary gel which correspond well to those obtained by IR spectroscopy results. The optical gap values (Table 1) were evaluated from the intercept of the linear portion of each curve hν in the X-axis. The obtained values for the optical band gap of the investigated samples varied depending on composition, and it was affected by the B_2_O_3_ content. It could be seen that sample A (25 mol% B_2_O_3_) exhibited a higher Eg value, which confirmed previous investigations obtained by other authors [62,63]. According to the literature data [62,63,64], the influence of B_2_O_3_ on the band gap value of TiO_2_ is very contradictory. Some authors found that when the boron content is below 10%, it causes a blue shift [18] while other scientific investigations summarized that even the addition of 5% B_2_O_3_ moved the TiO_2_ optical band gap to higher energies [62,63]. Therefore, it could be generalized that more experiments must be performed to clarify the role of boron in TiO_2_ optical properties. 

### 3.4. UV Photocatalytic Properties

**Figure 6:**
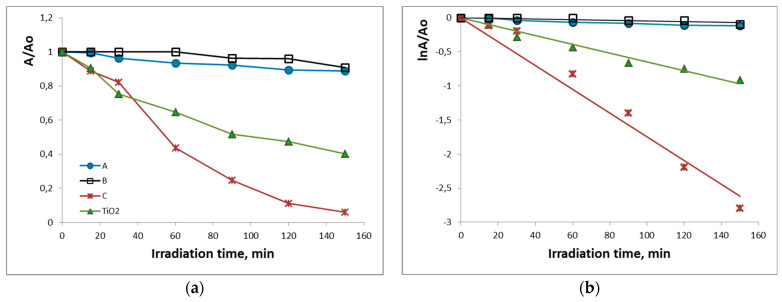
Photocatalytic activity of the synthesized samples heated at 500 °C for the degradation of MG under UV illumination: (**a**) change in absorbance as a function of irradiation time; (**b**) plot of lnA/Ao as a function of irradiation time.

The photocatalytic reaction was performed by using a model aqueous solution of Malachite green (MG) organic dye under UV irradiation (Figure 6a,b). It has to be noted that only heated at 500 °C samples were photocatalytically tested, and the obtained results were compared with those of pure TiO_2_ synthesized from Ti(IV) butoxide. As Figure 6a shows, the concentration of MG was reduced with the irradiation time to different degrees depending on the sample composition. Under UV irradiation for 150 min, the maximum degradation efficiency was achieved by the binary sample C (94%), greater than that of pure TiO_2_ (60%) and the ternary samples A (11%) and B (9%). The rate constants for the photodegradation of MG were calculated by the slopes of logarithmic plots lnA/Ao (Figure 6b). The highest rate constant (0.0174 min^−1^) was obtained for the sample 80TiO_2_/20B_2_O_3_ (C) followed by that of pure TiO_2_ (0.0065 min^−1^). Rate constants for the photodegradation of the ternary samples A (50TiO_2_.25TeO_2_.25B_2_O_3_) and B (80TiO_2_.10TeO_2_.10B_2_O_3_) were very low and similar (up to 0.0009 min^−1^).

Apparently, the highest MG decoloration was reached by the binary 80TiO_2_.20B_2_O_3_ sample which was higher than that of synthesized pure TiO_2_. Both ternary samples exhibited poorer but similar photocatalytic activity compared to the binary one. Up to now, the literature has summarized that the presence of boron promotes the formation of oxygen vacancies and photocatalytic properties [63,65,66]. To the best of our knowledge, the photocatalytic properties of TiO_2_/TeO_2_/B_2_O_3_ compositions have not been studied so far. Obviously, the addition of TeO_2_ did not improve the photocatalytic activity of the synthesized ternary samples. Bearing in mind that the photocatalytic activity depends on many factors [67], one of the possible reasons for the unsatisfying results could be the lower values of the surface area of the investigated samples. It is well known that TiO_2_ in the anatase phase is considered to show higher photocatalytic activity than TiO_2_ in an amorphous structure [67,68]. On the other hand, the lowest exhibited photocatalytic degradation efficiency of compositions A and B could be related to the XRD results and the presence of rutile as well as other crystalline phases (Figure 1). These results revealed that both the crystalline structure and surface area played a significant role in determining the photocatalytic efficiency of the synthesized powdered materials. 

### 3.5. Antibacterial Properties

**Figure 7:**
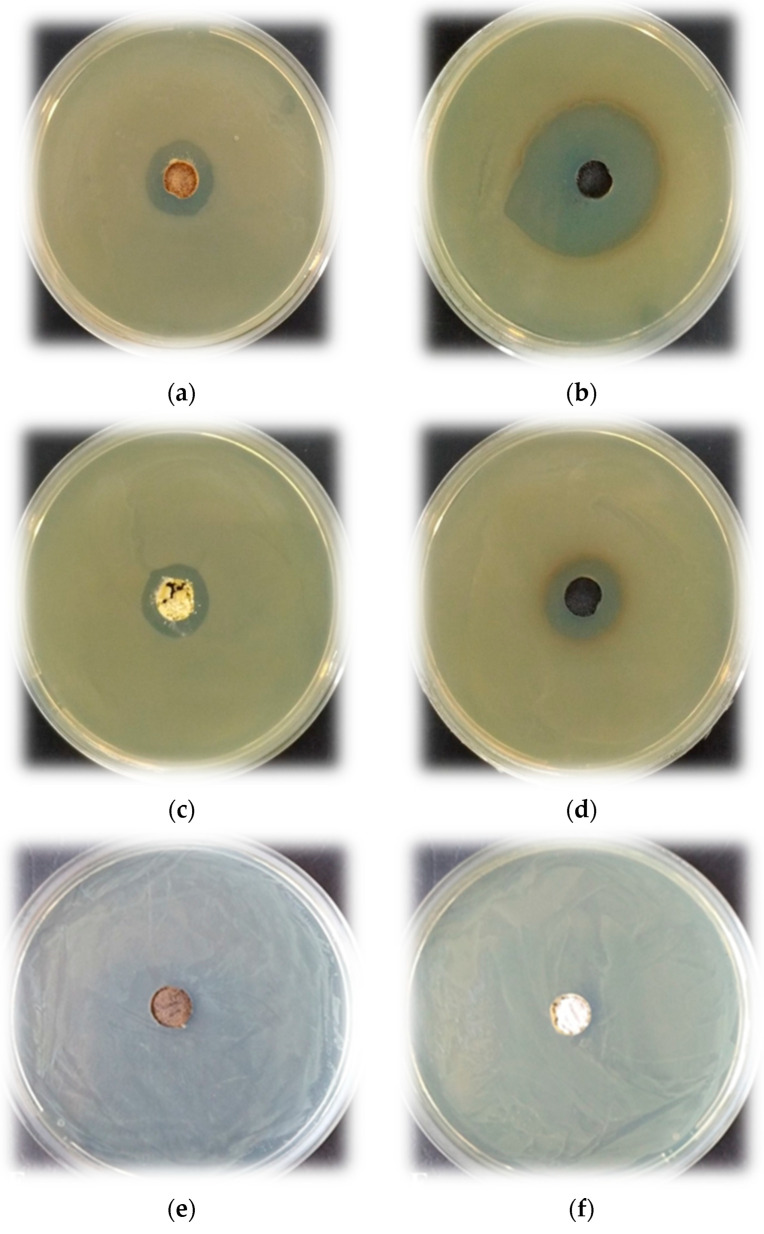
Antibacterial test result of the investigated samples heat-treated at 200 and 400 °C: (**a**,**b**) 50TiO_2_.25TeO_2_.25B_2_O_3_ (sample A); (**c**,**d**) 80TiO_2_.10TeO_2_.10B_2_O_3_ (sample B); and 80TiO_2_.20B_2_O_3_ (sample C) (**e**,**f**).

The antibacterial ability of the investigated samples calcined at 200 and 400 °C was tested against *E. coli* K12 NBIMCC 407 as a Gram-negative bacterium. The measurement of inhibition zones formed around the materials was the first method for the evaluation of the bactericidal effect of the investigated materials. All photographs of these investigations are given in Figure 7. It was observed that sample A (50TiO_2_.25TeO_2_.25B_2_O_3_) (Figure 7a,b) showed free growth zones against *E. coli* K12 407 with sizes 18.5 ± 0.25 mm and 36 ± 0.32 mm, at 200 and 400 °C, respectively. Obviously, the powder heat-treated at 400 °C (Figure 7b) inhibited to a great extent the studied strain compared to the sample heat-treated at 200 °C (Figure 7a). In comparison to these results, the other investigated sample B (Figure 7c,d) demonstrated relatively equal size zones for both temperatures, namely 17 ± 0.21 mm and 20 ± 0.27 mm. In this case, the processing temperature of the materials also affects the degree of inhibition of *E. coli* K12 407. The strain showed higher susceptibility to materials prepared at 400 °C (Figure 7d) and lower susceptibility to materials prepared at 200 °C (Figure 7c). In Figure 7e,f can be seen that the last sample C (80TiO_2_.20B_2_O_3_) exhibited formation of the smallest zones −11.5 ± 0.25 mm and 9.5 ± 0.19 mm. In contrast to the other samples (A and B), where the larger-size zones were observed, treated at 400 °C, this antibacterial test showed that the opposite tendency was established. 

The bactericidal effect of the investigated materials was also studied, calculating the percent of cell reduction after exposure of the strain to the materials. Concerning the first ternary sample A heated at 200 °C and 400 °C, the obtained results revealed the growth-inhibiting effect of the strain with 54% and 72% cell reduction, respectively. These values confirmed the above-discussed results from the agar-well diffusion test, namely that higher temperature exerts a stronger inhibitory effect on the strain. Similar to the results from the first antibacterial test where sample B at both temperatures demonstrated relatively equal size zones by using this approach, the same materials inhibit the cell growth with 52% and 56%, respectively. The binary sample C least affected the strain growth. The cell viability after treatment with powder heated at 200 °C was 62% and 70% after treatment with the sample heated at 400 °C. 

Generally, in the literature studies, the antibacterial activities of B_2_O_3_-containing compositions have great potential due to their promising biomedical applications. Moreover, within the last two decades, there has been increased interest in such compositions [4,65]. Aiming to interpret the discussed results, many factors influencing the antibacterial properties, such as specific surface area, morphology, size, etc., should be taken into consideration. Although all investigated samples possessed the average low value of the specific surface area (~35–45 m^2^/g), the inhibition of samples A and C against *E. coli* bacteria was higher than that of sample B as was described above. As seen in Figure 7, sample A contained a lower TiO_2_ amount (50 mol%), but it exhibited stronger antibacterial activity as compared to the other ternary sample B (80 mol% TiO_2_). According to the XRD results (Figure 1) at 400 °C, the presence of an amorphous part along with TiO_2_ (anatase) and metallic tellurium was detected in both samples A and B. Obviously, the higher B_2_O_3_ amount (25 mol%) in sample A is the reason for the increased antibacterial activity. For the binary sample C, it could be suggested that the synergistic effect of TiO_2_ and B_2_O_3_ provided efficient antibacterial activity. By analogy with our previous studies, we suggest that the antibacterial mechanism is realized through the formation of reactive oxygen species which decompose the outer membranes of bacteria and lead to cell death. Additionally, the IR spectroscopy showed that boron exists in the amorphous part of the samples most probably forming Ti–O–B bonds. According to Wang et al. [69], when boron atoms were incorporated into the TiO_2_ lattice, it enhanced the generation of the surface oxygen vacancies which improved the antimicrobial activity. The obtained results coincided with the literature studies [16,69,70,71]. It is well known that antimicrobial activity is an important factor in producing efficient nano-materials for sustainable applications. The obtained results give us reason to continue the investigations in this direction. Future studies will be performed aiming to improve the compositions and their efficiency for environmental applications.

## 4. Conclusions

Transparent gels containing B_2_O_3_ were obtained by applying a sol-gel method. They were calcined at different temperatures, and the results showed that above 300 °C, mixed powders of the amorphous phase, Te and TiO_2_ (anatase) were obtained. The IR data showed that the presence of H_3_BO_3_ decreases the rate of hydrolysis. The obtained UV-Vis spectra exhibited a red shift of the cut-off due to the presence of boron and tellurium units. The results from the photocatalytic tests for the degradation of Malachite green dye under UV irradiation revealed that the maximum photoactivity was achieved by the binary sample, whereas the photoactivity of the ternary samples was similar and very low, which could be explained by the effect of the crystalline structure and the low surface area. It was established that samples A and C which contain higher B_2_O_3_ content (above 20 mol%), exhibited higher antibacterial activity bearing in mind the percent of cell reduction after exposure to the *E. coli* K12 strain. However, more studies are needed to clarify the antimicrobial abilities of B-containing TiO_2_ nano-materials which will improve and enlarge their biomedical applications.

## Figures and Tables

**Table 1:** Cut-off and optical band gap values (Eg) of the investigated gel compositions.

Composition, mol%	Cut-off, nm	Eg, eV
Ti(IV) n-butoxide	389.71	3.18
TiO_2_	382.73	3.23
80TiO_2_.10TeO_2_.10B_2_O_3_ (sample B)	431.09	2.88
80TiO_2_.20B_2_O_3_ (sample C)	385.86	3.21
50TiO_2_.25TeO_2_.25B_2_O_3_ (sample A)	358.19	3.46

## Data Availability

Not applicable.
